# Targeted health and social care interventions for women and infants who are disproportionately impacted by health inequalities in high-income countries: a systematic review

**DOI:** 10.1186/s12939-023-01948-w

**Published:** 2023-07-11

**Authors:** Zahra Khan, Zoe Vowles, Cristina Fernandez Turienzo, Zenab Barry, Lia Brigante, Soo Downe, Abigail Easter, Seeromanie Harding, Alison McFadden, Elsa Montgomery, Lesley Page, Hannah Rayment-Jones, Mary Renfrew, Sergio A. Silverio, Helen Spiby, Nazmy Villarroel-Williams, Jane Sandall

**Affiliations:** 1 Department of Women & Children’s Health, King’s College London, London, UK; 2 Patient and Public Involvement and Engagement, NIHR ARC South London, London, UK; 3 Royal College of Midwives, London, UK; 4 University of Central Lancashire, Lancashire, UK; 5 Department of Population Health Sciences, King’s College London, London, UK; 6 School of Health Sciences, University of Dundee, Dundee, UK; 7 Methodologies Division, King’s College London, London, UK; 8 King’s College London, London, UK; 9 University of Dundee, Dundee, UK; 10 University of Nottingham, Nottingham, UK; 11 Sheffield Hallam University, Sheffield, UK

**Keywords:** Health inequality, Targeted intervention, High-income country, Midwife models, Interdisciplinary care, Community care, Disadvantage, Social complexity, Ethnic minority

## Abstract

**Background:**

Disadvantaged populations (such as women from minority ethnic groups and those with social complexity) are at an increased risk of poor outcomes and experiences. Inequalities in health outcomes include preterm birth, maternal and perinatal morbidity and mortality, and poor-quality care. The impact of interventions is unclear for this population, in high-income countries (HIC). The review aimed to identify and evaluate the current evidence related to targeted health and social care service interventions in HICs which can improve health inequalities experienced by childbearing women and infants at disproportionate risk of poor outcomes and experiences.

**Methods:**

Twelve databases searched for studies across all HICs, from any methodological design. The search concluded on 8/11/22. The inclusion criteria included interventions that targeted disadvantaged populations which provided a component of clinical care that differed from standard maternity care.

**Results:**

Forty six index studies were included. Countries included Australia, Canada, Chile, Hong Kong, UK and USA. A narrative synthesis was undertaken, and results showed three intervention types: midwifery models of care, interdisciplinary care, and community-centred services. These intervention types have been delivered singularly but also in combination of each other demonstrating overlapping features. Overall, results show interventions had positive associations with primary (maternal, perinatal, and infant mortality) and secondary outcomes (experiences and satisfaction, antenatal care coverage, access to care, quality of care, mode of delivery, analgesia use in labour, preterm birth, low birth weight, breastfeeding, family planning, immunisations) however significance and impact vary. Midwifery models of care took an interpersonal and holistic approach as they focused on continuity of carer, home visiting, culturally and linguistically appropriate care and accessibility. Interdisciplinary care took a structural approach, to coordinate care for women requiring multi-agency health and social services. Community-centred services took a place-based approach with interventions that suited the need of its community and their norms.

**Conclusion:**

Targeted interventions exist in HICs, but these vary according to the context and infrastructure of standard maternity care. Multi-interventional approaches could enhance a targeted approach for at risk populations, in particular combining midwifery models of care with community-centred approaches, to enhance accessibility, earlier engagement, and increased attendance.

**Trial registration:**

PROSPERO Registration number: CRD42020218357.

**Supplementary Information:**

The online version contains supplementary material available at 10.1186/s12939-023-01948-w.

## Background

High-income countries (HICs) [1] have comparatively lower rates of maternal and perinatal mortality than low- and middle-income countries (LMICs); however, outcomes vary between and within countries [2]. Within the United Kingdom (UK), for example, there are differences in mortality, morbidity and experiences of maternity care [3–5]. Those living in the most deprived areas of the UK are more likely to experience a stillbirth, neonatal death, preterm birth and maternal mortality [6]. Also, the rate of stillbirth, neonatal mortality, and maternal mortality, are disproportionately higher for minority ethnic groups [6, 7]. These measurable differences in experience and outcomes are known as health inequalities, [8]. Health inequalities are avoidable and are the result of unequal distribution of resources, power, and income in society [9].

Some population groups at higher risk of health inequalities are often described in the literature as vulnerable or having social risk factors [10]. This is known to include women who experience multiple and severe social disadvantages, including but not limited to: homelessness, poverty, domestic violence, substance misuse, or those from minority ethnic groups [11]. Unlike LMICs, HICs have infrastructure, resources, and finances available, but are still failing populations that are disproportionately at risk of inequalities, and in some cases, the inequality gap has been widening [12]. Equally, it is also important to know which interventions work and to build on their strengths [13].

Health and social care interventions aim to improve the health and wellbeing of their targeted populations. Interventions range from: surgical and pharmacological, health promotion and education, immunisation campaigns, financial subsidies, and upskilling professionals and models of care [14]. Models of care are complex interventions, with various interacting components and mechanisms [15], and are commonly used in health and social care. However, universal interventions, targeting whole populations, are not ideal for addressing specific health inequalities and have also been shown to widen inequalities [14]. The Strategic Review of Health Inequalities in England introduced the concept of ‘proportionate universalism’ [16] to this debate, suggesting that health actions must be universal, not targeted (to avoid stigmatisation), but with a scale and intensity that is proportionate to the level of disadvantage.

There is evidence available for the benefits of healthy women receiving different models of maternity care interventions, for example, community-based [17] and midwife-led or doctor-led care [18, 19]. Some evidence of targeted interventions for vulnerable women has included different stages of maternity care, for example, antenatal programmes for women with social complexities or women living in deprived areas [20, 21] but few have included all areas of maternity care [21]. There is also a review of interventions which reduce health inequalities in LMICs [14], however there is no similar evidence for HICs. To date, there has not been a comprehensive review of targeted models of care interventions including all areas of maternity care (antenatal; intrapartum; postnatal) in HICs. This review aims to systematically identify and evaluate the current evidence available related to targeted health and social care interventions in HICs to reduce health inequalities experienced by disproportionately at-risk women and infants.

## Methods

The protocol of this review was registered and published with PROSPERO (CRD42020218357) [22]. The review followed the Preferred Reporting Items for Systematic Reviews and Meta-Analyses-Equity (PRISMA-E) reporting guidelines [23] (see additional file 1). A mixed-methods approach was taken to consider a breadth of research designs that included complex health and social interventions. This would allow for a comprehensive understanding of what works, what doesn’t work, how and in what contexts.

### Search strategy and study selection

An electronic search strategy was undertaken using 12 health-related databases (MEDLINE, EMBASE, PsychInfo, MIDIRS, Global Health, BNI, Web of Science, CINAHL, CENTRAL, LILACS, AJOL, Global Index Medicus). Further to this JBI and other systematic reviews, national and international reports, dissertation and theses, grey literature, ISRCTN registry, PROPERO, Cochrane, and the Australian and the New Zealand Clinical Trials Registry were also searched. Lastly, a backward hand-search of bibliographies and reference lists of the included studies was also undertaken. The setting, perspective, intervention, comparison, and evaluation (SPICE) question framework was used to develop the research question and identify a complete list of keywords (see additional file 2) for the search.

Eligibility criteria were developed (see additional file 3). No study design, date of publication, or language restrictions were applied. The search included all HICs as defined by the 2019 World Bank Gross National Income (GNI) [1]. The population included childbearing women, newborns, and infants up to one year of age who are deemed by predetermined criteria [6, 11, 24, 25] as disproportionately impacted by health inequalities (see Table 1). The search for publications ended on 08/11/2022.

**Table 1:** Characteristics of populations at risk of health inequalities [6, 11, 24, 25]

Women who find services hard to access	Women needing multiagency services
• Ethnic minority or Indigenous people • Socially isolated women • Those living in poverty/deprivation/who are homeless • Refugees/asylum seekers • Non-native language speakers • Victims of abuse • Sex workers • Young mothers • Unsupported mothers • Women within travelling communities (Gypsy, Traveller and Roma)	• Women who are subject of safeguarding concerns • Women with substance and/or alcohol abuse issues • Women with physical/emotional and/or learning disabilities • Women who have been victims of female genital mutilation • Women who are HIV positive

The intervention criteria were defined as any health or social care intervention which included clinical care as part of the programme or package of care, which was different from the setting's standard care. Standalone interventions (e.g., vouchers, supplements), interventions which did not include clinical care (peer support), adjuncts to existing care or any interventions that were not part of an overall programme or package of care (e.g., educational class), and well-established targeted interventions with existing Cochrane reviews (e.g., family nurse partnership, social support) [26–28]were excluded from this review.

Primary outcomes were maternal, perinatal, and infant mortality, and secondary outcomes included experiences and satisfaction, antenatal care coverage, access to care, quality of care, mode of delivery, analgesia use in labour, preterm birth, low birth weight, breastfeeding, family planning, immunisations (see additional file 4 for outcome definitions). Studies were included irrespective of whether the intervention had been identified as a success, to help meet the objectives of this review and understand what does, or does not, work. Inequality indicators (e.g., differences between sample groups based on sociodemographic, ethnicity, race, deprivation index, or others described by the authors of papers) were reported and discussed in relation to the outcomes.

### Study selection, quality assessment and data extraction

Covidence was used to manage the screening and study selection process. All papers were screened by title and abstract by a first and second reviewer and any conflicts were discussed and agreed upon with a third reviewer. The methodological quality of included papers was assessed using the Mixed Methods Appraisal Tool (MMAT) [29], as it offers assessment of quantitative (randomised, non-randomised, descriptive), qualitative and mixed methods design. The tool offers three response options: ‘Yes’ the criterion is met, ‘No’ the criterion is not met, and ‘Can’t tell’ when there is not enough information to judge. The updated 2018 version of MMAT [29] advises against scoring criteria as it does not provide enough detail, however if required scores are determined based on how many of the criteria is met. For example, ***** for 100%, **** 80%, *** 60%, ** 40%, * ≤ 20% of the “yes” criteria have been met. See Table 2 for overall MMAT scores. A pre-designed data extraction form was piloted and used to extract study characteristics and outcome data, initially in Covidence and then tabulated in Excel. Quality assessment and data extraction were assessed independently by a first and a second reviewer and any conflicts were discussed and consensus reached with a third reviewer.

**Table 2:** Summary of studies included

Author, Date	Country	Design	Population	Intervention	Primary Outcomes	Secondary Outcomes	MMAT score
Allen 2016 [30]	Australia	Mixed methods	Young mothers	Midwifery models		Experience/ Satisfaction; Antenatal care coverage; Access to care; Quality of care	^b^
Alliman 2019 [31]	USA	Qualitative research	Socially isolated women; Those living in poverty/ deprivation/ who are homeless; Unsupported mothers; Ethnic minorities	Midwife models		Experience/ Satisfaction; Family planning; Breastfeeding; Low birth weight; Preterm birth; Analgesia use; Mode of birth; Access to care	^a^
Alvarado 1999 [32]	Chile	Mixed methods	Those living in poverty/ deprivation/ who are homeless	Interdisciplinary care	Maternal mortality	Experience/ Satisfaction; Family planning; Breastfeeding; Access to care	^e^
Balaam 2018 [10]	UK	Qualitative research	Socially isolated women; Unsupported mothers; Women who are subject of safeguarding concerns	Midwifery models		Experience/Satisfaction	^a^
Barkauskas 2002 [33]	USA	Non-randomised studies	Socially isolated women; Those living in poverty/ deprivation/ who are homeless; Unsupported mothers; Women with substance and/or alcohol abuse issues	Midwifery models		Breastfeeding; Low birth weight; Preterm birth; Mode of birth	^c^
Bertilone, 2015 [34]	Australia	Non-randomised studies	Those living in poverty/ deprivation/ who are homeless; Young mothers; Ethnic minorities	Midwifery models		Low birth weight; Preterm birth; Mode of birth; Access to care	^b^
Blanchette 1995 [35]	USA	Non-randomised studies	Those living in poverty/ deprivation/ who are homeless; Women with substance and/or alcohol abuse issues; Ethnic minorities; Other: Low income uninsured and underinsured women in the USA	Midwifery models	Perinatal mortality	Low birth weight; Preterm birth; Analgesia use; Mode of birth; Access to care	^d^
Campbell 2004 [36]	Australia	Non-randomised studies	Ethnic minorities	Midwifery models; Community-centred		Experience/ Satisfaction; Breastfeeding; Access to care; Quality of care	^b^
Cunningham 2017 [37]	USA	Randomised controlled trial	Young mothers; Ethnic minorities	Midwifery models		Experience/Satisfaction; Antenatal care coverage	^d^
Filby 2019 [42[	UK	Qualitative research	Ethnic minorities	Midwifery models		Experience/Satisfaction; Antenatal care coverage; Quality of care	^a^
Grady 2004 [38]	USA	Non-randomised studies	Young mothers; Ethnic minorities	Midwifery models		Experience/ Satisfaction; Breastfeeding; Low birth weight; Preterm birth; Mode of birth; Antenatal care coverage; Access to care	^d^
Homer 2012 [39]	Australia	Mixed methods	Ethnic minorities	Midwifery models	Perinatal mortality	Experience/ Satisfaction; Breastfeeding; Low birth weight; Preterm birth; Mode of birth; Access to care	^a^
Ip 2015 [40]	Hong Kong	Non-randomised studies	Socially isolated women Young mothers; Unsupported mothers; Women with substance and/or alcohol abuse issues	Interdisciplinary care		Experience/Satisfaction	^a^
Jan 2004 [41]	Australia	Mixed methods	Socially isolated women; Those living in poverty/ deprivation/ who are homeless; Unsupported mothers; Ethnic minorities	Midwifery models; Community-centred	Perinatal mortality	Experience/ Satisfaction; Low birth weight; Antenatal care coverage; Access to care	^b^
Jones 2021 [42]	UK	Quantitative descriptive	Those living in poverty/ deprivation/ who are homeless; Young mothers; Unsupported mothers; Women with substance and/or alcohol abuse issues	Midwifery models; Interdisciplinary care		Breastfeeding; Antenatal coverage; Access to care	^c^
Kildea 2012 [43]	Australia	Mixed methods	Ethnic minorities	Midwifery models; Interdisciplinary care; Community-centred		Experience/Satisfaction; Breastfeeding; Low birth weight; Preterm birth; Analgesia use; Mode of birth; Antenatal care coverage; Access to care; Quality of care	^a^
Kildea 2019 [44]	Australia	Non-randomised studies	Socially isolated women; Ethnic minorities	Midwifery models		Preterm birth; Antenatal care coverage; Access to care	^a^
Kildea 2021 [45]	Australia	Non-randomised studies	Ethnic minorities	Midwifery models; Community-centred		Breastfeeding; Low birth weight; Preterm birth; Analgesia use; Mode of birth; Antenatal coverage; Access to care	^a^
Klerman 2001 [46]	USA	Randomised controlled trial	Ethnic minorities; Other: Medicaid women (low income families)	Midwifery models		Experience/Satisfaction; Low birth weight; Preterm birth; Mode of birth; Antenatal care coverage; Quality of care	^c^
Lack 2016 [47]	Australia	Quantitative descriptive studies	Women who have been victims of female genital mutilation; Ethnic minorities; Other: Most communities are socio-economic disadvantaged and housing and infrastructure managed by government	Midwifery models	Perinatal mortality	Low birth weight; Preterm birth; Mode of birth; Antenatal care coverage; Access to care	^b^
Lenaway 1995	USA	Non-randomised studies	Those living in poverty/ deprivation/ who are homeless; Other: recipients of Medicaid or the Colorado Indigent Care Program	Midwifery models		Low birth weight; Preterm birth; Mode of birth; Access to care	^b^
Liu 2017 [48]	USA	Mixed methods	Socially isolated women; Those living in poverty/ deprivation/ who are homeless; Non-native language speakers; Ethnic minorities	Midwifery models		Experience/Satisfaction	^d^
Madeira 2019 [49]	USA	Mixed methods	Non-native language speakers; Ethnic minorities	Midwifery models		Experience/Satisfaction	^b^
Malebranche 2020 [50]	Canada	Non-randomised studies	Refugees/asylum seekers	Interdisciplinary care		Low birth weight; Preterm birth; Mode of birth; Antenatal care coverage; Quality of care	^a^
McAree 2010 [51]	UK	Qualitative research	Ethnic minorities	Midwifery models		Experience/Satisfaction	^a^
Mersky 2021	USA	Randomised controlled trial	Those living in poverty/ deprivation/ who are homeless	Community-centred		Breastfeeding	^d^
Middleton 2017 [52]	Australia	Mixed methods	Those living in poverty/ deprivation/ who are homeless; Young mothers; Unsupported mothers; Ethnic minorities	Midwifery models; Community-centred		Experience/ Satisfaction; Breastfeeding; Low birth weight; Preterm birth; Antenatal care coverage; Access to care	^a^
Morris 2012 [53]	Australia	Qualitative research	Women with substance and/or alcohol abuse issues	Interdisciplinary care		Experience/Satisfaction	^a^
Nel 2003 [54]	Australia	Quantitative descriptive studies	Socially isolated women; Those living in poverty/ deprivation/ who are homeless; Ethnic minorities	Midwifery models	Maternal mortality	Antenatal care coverage	^e^
Owens 2016 [55]	Australia	Qualitative research	Refugees/asylum seekers; Non-native language speakers; Ethnic minorities	Midwifery models; Community-centred		Experience/Satisfaction	^a^
Panaretto 2005	Australia	Non-randomised studies	Ethnic minorities	Midwifery models; Community-centred	Perinatal mortality	Low birth weight; Preterm birth; Antenatal care coverage; Access to care; Quality of care	^c^
Piechnik 1985 [56]	USA	Non-randomised studies	Those living in poverty/ deprivation/ who are homeless; Young mothers; Ethnic minorities	Interdisciplinary care	Maternal mortality; Perinatal mortality	Family planning; Breastfeeding; Immunisation; Mode of birth	^c^
Quelly 2021 [57]	USA	Quantitative descriptive studies	Those living in poverty/ deprivation/ who are homeless; Other: recipients of Medicaid	Interdisciplinary care		Low birth weight; preterm birth; Antenatal coverage; Access to care	^d^
Quinlivan 2003 [58]	Australia	Randomised controlled trial	Socially isolated women; Those living in poverty/ deprivation/ who are homeless; Young mothers; Women with substance and/or alcohol abuse issues; Ethnic minorities	Midwifery models	Perinatal mortality	Family planning; Breastfeeding; Immunisation; Mode of birth; Access to care	^c^
Reeve 2016 [59]	Australia	Non-randomised studies	Those living in poverty/ deprivation/ who are homeless; Ethnic minorities	Midwifery models; Interdisciplinary care; Community-centred	Perinatal mortality	Low birth weight; Preterm birth; Mode of birth; Antenatal care coverage; Access to care	^c^
Reguero 1994 [60]	USA	Quantitative descriptive studies	Those living in poverty/ deprivation/ who are homeless; Women with substance and/or alcohol abuse issues; Ethnic minorities	Interdisciplinary care; Community-centred	Infant mortality		^d^
Robertson 2009 [61]	USA	Non-randomised studies	Ethnic minorities	Midwifery models		Experience/ Satisfaction; Breastfeeding; Preterm births; Mode of birth	^e^
Ross 1981 [62]	USA	Quantitative descriptive studies	Ethnic minorities; Other: Indian American tribe	Midwifery models	Perinatal mortality	Preterm birth; Antenatal care coverage; Access to care	^d^
Rutman 2020 [63]	Canada	Mixed methods	Socially isolated women; Those living in poverty/ deprivation/ who are homeless; Victims of abuse; Young mothers; Women with substance and/or alcohol abuse issues	Interdisciplinary care		Experience/ Satisfaction; Access to care	^e^
Smoke 1988 [64]	USA	Non-randomised studies	Young mothers	Midwifery models; Interdisciplinary care		Family planning; Breastfeeding; Low birth weight; Preterm birth; Analgesia use; Mode of birth; Antenatal care coverage; Access to care	^a^
Stapleton 2013 [65]	Australia	Mixed methods	Socially isolated women; Refugees/asylum seekers; Non-native language speakers; Ethnic minorities	Midwifery models; Interdisciplinary care		Experience/ Satisfaction; Breastfeeding; Preterm birth; Mode of birth	^d^
Tandon 2013 [66]	USA	Non-randomised studies	Non-native language speakers; Ethnic minorities	Midwifery models		Experience/ Satisfaction; Antenatal care coverage	^b^
Trudnak 2017	USA	Non-randomised studies	Non-native language speakers; Ethnic minorities	Midwifery models		Breastfeeding; Low birth weight; Preterm birth; Mode of birth; Access to care	^b^
Turner 2000 [67]	USA	Quantitative descriptive studies	Women who are HIV positive	Interdisciplinary care		Experience/Satisfaction; Low birth weight; Preterm birth; Access to care; Quality of care	^c^
Wiggins 2005 [68]	UK	Randomised controlled trial	Those living in poverty/ deprivation/ who are homeless; Non-native language speakers	Midwifery models		Experience/ Satisfaction; Breastfeeding; Antenatal care coverage; Access to care	^c^
Wong 2011 [69]	Australia	Quantitative descriptive studies	Ethnic minorities	Midwifery models	Perinatal mortality; Infant mortality	Low birth weight; Preterm birth; Mode of birth; Antenatal care coverage; Access to care	^a^

“yes” criteria have been met

^a^ for 100%,

^b^ for 80%,

^c^ for 60%,

^d^ for 40%,

^e^ for ≤ 20% of MMAT

The quality of each study was evaluated against two screening questions and five further questions relating to their design, unless they were of mixed method designs in which case they had a further fifteen questions.

### Data analysis

Due to the wide variations in research designs, intervention types and outcomes a meta-analysis could not be performed. Instead, results are presented narratively and organised based on the interpreted intervention types.

## Results

**Fig. 1:**
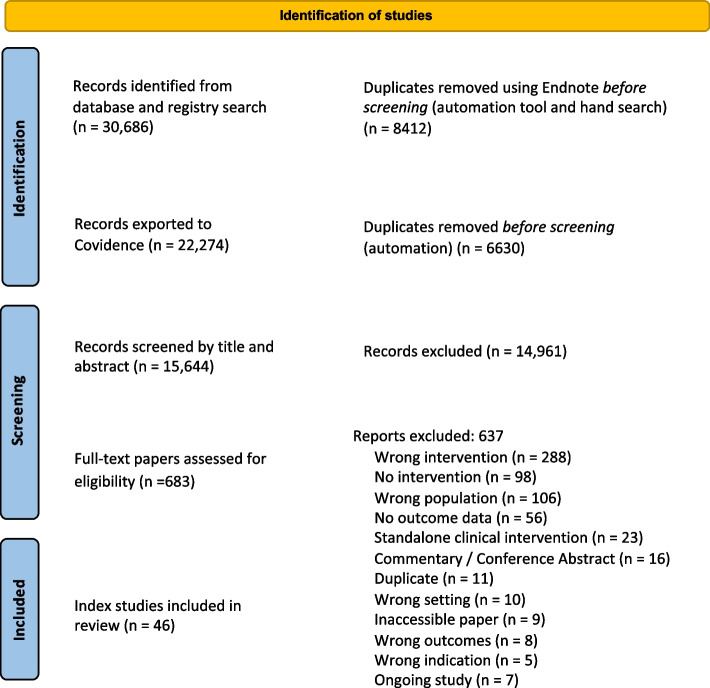
PRISMA flow diagram

The initial database and hand search resulted 30,686 references. After duplicates were removed, 15,644 papers were screened by title and abstract, and 683 full text papers were screened for inclusion. Finally, 53 papers were included, some of which were merged with their index papers, resulting in 46 studies included in the review (see Fig. 1).

### Methodological characteristics and quality of included studies

The 46 included index studies varied in assorted methodological design: mixed methods (*n* = 10), qualitative (*n* = 6), quantitative randomised control trial (*n* = 5), quantitative non-randomised (*n* = 17), quantitative descriptive (*n* = 8). The high-income countries included Australia (*n* = 18 + 3 sibling papers), Canada (*n* = 2), Chile (*n* = 1), Hong Kong (*n* = 1), UK (*n* = 5) and USA (*n* = 19 + 4 sibling papers). From the disadvantaged population groups (see Table 1) all were identified except 3 categories (sex workers; travelling communities; physical/emotional/learning disabilities) (see Table 3). Categories of population groups were not homogenous and often intersected demonstrating multiple disadvantages. The earliest study was published in 1981 and the most recent study was published in 2021. No study was excluded based on their score as recommended by MMAT tool.

**Table 3:** Populations studied across interventions

Population characteristics	Midwifery models	Interdisciplinary care	Community-centred
Socially isolated women	**✓**	**✓**	**✓**
Living in poverty/deprivation or homeless	**✓**	**✓**	**✓**
Refugees/asylum seekers	**✓**	**✓**	**✓**
Non-native language speakers	**✓**	**✓**	**✓**
Victims of abuse		**✓**	**✓**
Sex workers			
Young mothers	**✓**	**✓**	**✓**
Unsupported mothers	**✓**	**✓**	**✓**
Women within travelling communities			
Safeguarding concerns	**✓**		
Women with substance/alcohol abuse issues	**✓**	**✓**	**✓**
Physical/emotional/learning disabilities			
Victims of female genital mutilation	**✓**		
Women who are HIV positive		**✓**	
Ethnic minorities	**✓**	**✓**	**✓**

### Interventions

**Fig. 2:**
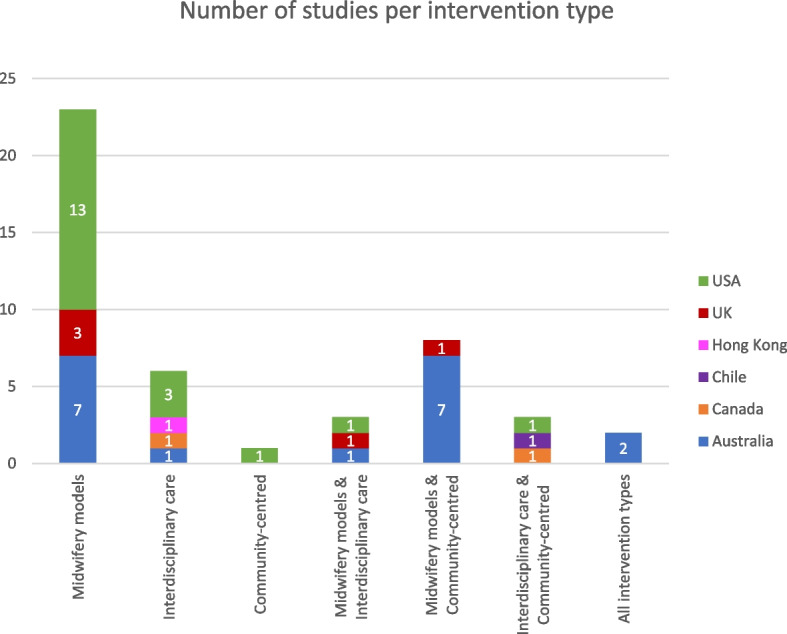
Number of studies per intervention type

Upon narrative synthesis [70] three principal interventions were identified: midwifery models of care; interdisciplinary care; and community-centred services. The principal interventions were synthesised by grouping common features of the interventions such as, how they were delivered, the main clinician, and how they were managed and organised. The interventions were not mutually exclusive, and in some studies a multi-intervention approach was taken (see Fig. 2), combining with another intervention type. Two studies [43, 59] combined all three intervention categories.

Midwifery models of care took an interpersonal and holistic approach as they focused on continuity of carer, home visiting, culturally and linguistically appropriate care and accessibility (financial and geographic). For example, studies in this intervention type considered interpersonal relationships between women, including families and communities, and the health care professional who was typically a midwife, or similarly qualified depending on the context. Some studies also considered wider relationships in group antenatal care settings. Qualitative results strongly suggested the importance of interpersonal relationships in women’s overall experiences. Interdisciplinary care took a structured approach, to coordinate care between different health and social care services for women. Studies which researched this intervention predominantly focused on complexities such as HIV, substance and/or alcohol misuse, and those living in deprivation, as they required multi-agency services. Community-centred services took a community-based approach with interventions that suited the need of its community and their specific norms, particularly ethnic minority populations or those with non-native language speaking ability. Studies based in Australia also included community members and/or health care practitioners from the same ethnic/cultural background as the population, which was overall positively evaluated. Most community-centred interventions were multi-interventional combining with predominantly midwifery models of care and in some instance interdisciplinary care.

Results are presented in three sections based on each intervention, rather than just primary and secondary outcomes. The purpose of this is to help readers understand the specificity of intervention types, followed by outcome indicators and patterns across contexts.

### Midwifery models of care

Midwifery models of care were defined by the review team as interventions with midwives, or those similarly qualified based on the setting, as the central care providers or coordinators of care. There is often continuity from the care provider, and/or care is shared in a caseload. Midwifery models can also include shared care between a midwife and a primary physician or general practitioner, who is available for escalation and/or also provides regular care. The format of antenatal care is either individual or in a group a group setting. Overall, 36 studies incorporated midwifery models of care interventions. Countries included Australia (*n* = 17), UK (*n* = 5) and USA (*n* = 14). This intervention was the most frequently reported. Twenty-three studies were exclusively midwifery models of care studies [10, 30, 31, 33, 35, 37–39, 44, 46–49, 51, 54, 58, 61, 62, 66, 68, 69, 71–73]. Three studies combined midwifery models and interdisciplinary care interventions [42, 64, 65], whereas eight studies combined midwifery models with community-centred services [34, 36, 41, 42, 45, 52, 55, 74]. Two studies combined all three interventions [43, 59].

#### Primary outcomes

Maternal mortality was reported in one study’s intervention as lower [54]. Perinatal mortality (stillbirth or neonatal death) was reported in nine studies, either as not statistically significant [41, 62, 74], lower in the intervention group [58], or reported without a comparator making it difficult to draw conclusions [35, 39, 47, 58, 59, 69]. Infant mortality was reported in one study [69].

#### Secondary outcomes

Twenty studies included results of participants experiences which were overall positive. Frequently cited reasons for positive experiences included feeling informed and having information explained [31, 36, 41, 46, 49, 51, 55], having more time in their appointments [31, 36, 41, 46, 66] and better access to their midwife [41, 71], having trust and being treated with respect [10, 31, 36, 39, 41, 48, 49, 71] as well as family centred social support [10, 30, 31, 41, 55]. Women also valued knowing who their care provider was [30, 36, 51], and particularly appreciated continuity of care from their midwife [39, 41, 43, 51, 65, 71]. In addition to this, women emphasised the value of receiving care from a midwife similar to their ethnic background [52] or a bilingual practitioner [48, 66]. Care in a community and/or group setting was viewed positively [39, 48, 49, 51, 61, 66] especially by adolescent groups [30, 37, 38] as they enjoyed interacting with others in similar situations to them therefore feeling less isolated. However, some did not find care in a group culturally appropriate [68] and some non-health community settings, such as immigration accommodation centre, were not fit for purpose but outside the control of maternity services [71].

There was increased knowledge and use of contraception in the intervention groups [31, 58] and also a variety of contraception methods were utilised [64]. Breastfeeding rates were frequently higher in the intervention groups [31, 33, 36, 38, 39, 42, 45, 52, 64, 68], in some instances similar or no differences were reported [43, 58, 61, 65]. One study reported no differences in immunisation knowledge or uptake [58].

There were lower rates of preterm birth [31, 34, 38, 44–47, 62, 64, 69, 72, 74] and low birth weight [31, 34, 38, 47, 64, 69, 72], however, some studies found no difference or no statistical significance for preterm birth [33, 35, 39, 43, 52, 59, 65, 73] rates and low birth weight [33, 35, 39, 41, 43, 52, 59, 73, 74]. Some reasons for lack of significance included small sample sizes.

Overall, mode of birth was positively reported with higher rates of spontaneous vaginal birth [43, 58, 61, 65, 73] and lower rates of caesarean Sect. [31, 33, 35, 43, 45, 46, 58, 69, 73] in the intervention groups. Again, in some instances study findings showed no significant differences [34, 38, 47, 59, 64, 72]. It was also noted that there was less use of epidural analgesia in the intervention groups [35, 43, 45]. Intervention groups were found to have earlier first-trimester appointments [30, 31, 34, 39, 41, 43, 44, 47, 52, 58, 59, 62, 69, 74] and higher rates of antenatal care coverage [30, 41–46, 52, 54, 62, 64, 69, 74]. They were also more likely to accept referrals [30] and have a documented care plan [74].

#### Patterns in countries

Midwifery models of care interventions were the most cited intervention type with three subgroups: continuity of care, shared care and group antenatal care. From the countries included in this review, Canada, Chile and Hong Kong did not incorporate midwifery models of care interventions. Australian studies often combined this intervention with community-centred service interventions and were predominantly targeting Aboriginal communities [34, 36, 41, 43, 45, 52, 55, 59, 74]. In this context, the specific model included continuity of care by a midwife and/or shared care with a doctor (general practitioner or obstetrician). Continuity of care was also implemented in the USA and UK interventions either by a midwife or certified nurse-midwife [10, 33, 46, 51, 62, 71]. Group antenatal care provided by midwives in the USA and Australia were targeting either adolescent pregnancies [30, 37, 38] or ethnic minority populations [48, 49, 61, 73].

### Interdisciplinary care

Interdisciplinary care interventions were defined as care requiring multi-service involvement, whereby care is provided by a range of health and/or social care professionals, beyond the services of standard care. Overall, fourteen studies incorporated interdisciplinary care interventions and professionals included midwives, obstetricians, nurses, paediatricians, social workers, psychologists, dieticians, nutritionists and pharmacists. Countries included Australia (*n* = 4), Canada (*n* = 2), Chile (*n* = 1), Hong Kong (*n* = 1), UK (*n* = 1) and USA (*n* = 5). Three studies combined interdisciplinary care with midwifery models of care [42, 64, 65], three studies with community-centred services [32, 60, 63], and two studies combined all three intervention groups [43, 59]. Six studies were exclusively interdisciplinary care interventions [40, 50, 53, 56, 57, 67].

#### Primary outcomes

Four studies reported primary outcomes [32, 56, 59, 60]. One study [32] reported one maternal mortality from the intervention group, however there was no further statistical analysis regarding significance. Two studies reported perinatal mortality, one of which reported lower rates in the intervention group [56], however the other study [59] was not statistically significant. One study [60] reported infant mortality decreased, however this could not be directly linked to the intervention.

#### Secondary outcomes

Seven studies [32, 40, 43, 53, 63, 65, 67] included experiential outcomes. Overall, intervention groups reported higher levels of satisfaction [32, 40, 43, 65, 67], citing reasons as having time to ask questions, continuity, being treated with respect. However, women were disappointed with the lack of continuity in labour and the postnatal period [43, 65], and reported some staff imposing control over their care [53, 65]. Higher rates of antenatal care coverage and of first-trimester initial appointments were reported in the intervention groups [42, 43, 57, 59, 64], however one study found that the intervention group of asylum-seeking women took significantly longer to present and attended less visits [50].

Higher rates of vaginal birth and lower caesarean section rates were found in the intervention [43, 64, 65] but in some cases findings were not significant in certain studies [50, 56, 59]. Use of epidural analgesia was lower in one [43] instance but higher in another [56]. Preterm birth rates varied, as they were lower in some intervention groups [64, 65, 67], not statistically significant in others [43, 59] and in one retrospective study noted as higher than the national average [50]. Lower rates of low birth weight were reported in the intervention groups in four studies [43, 56, 64, 67] and in one study were not statistically significant [59].

Three studies noted higher rates of breastfeeding in the intervention groups [32, 42, 64], however another two studies reported similar rates [43, 65]. Intervention groups were noted to use a wider range of postnatal contraceptives compared to comparison groups [32, 64]. Contraception initiation in one study [32] was similar in the intervention and comparison group. Reasons for higher rates of no contraception use in the intervention group were because of not having a current partner and therefore not required. There was no immunisation data for interdisciplinary care models.

#### Patterns in countries

Interdisciplinary care interventions were the second most common intervention type found in this review and the only intervention type found for Hong Kong (*n* = 1). Outcomes varied between interventions therefore it is difficult to conclude any patterns. Types of health and social care professionals varied between studies and were specific to the population needs. For example, three studies [53, 60, 63] targeted women with substance misuse however they all had different professionals address these needs from obstetricians to counsellors. Having said that, both midwives and doctors (obstetrician, general physician and/or paediatrician) were the primary professionals cited as part of the interdisciplinary care interventions.

### Community-centred services

Community-centred service interventions were defined as services that were addressing the specific needs of their population and/or implementing a public health model in a community setting. Overall, fourteen studies incorporated community-centred interventions and countries included Australia (*n* = 9), Canada (*n* = 1), Chile (*n* = 1), UK (*n* = 1) and USA (*n* = 2). This intervention was not implemented independently in any of the 14 studies. Almost all community-centred interventions were in combination with another intervention type (midwifery model or interdisciplinary care), except for one study in the USA [75]. Combined midwifery models of care and community-centred services were reported in eight studies [34, 36, 41, 45, 52, 55, 71, 74]. Combined interdisciplinary care and community-centred services were reported in three studies [32, 60, 63]. Two studies combined all three interventions [43, 59].

#### Primary outcomes

Five studies [32, 41, 59, 60, 74] reported primary outcomes. One study reported one maternal mortality [32] but again did not state statistical significance. Perinatal mortality was reported by three studies [41, 59, 74] none of which were statistically significant. Neonatal mortality reported by one study [60] found a reduction in mortality over a two-year period however it was unclear if it was a direct result of the intervention.

#### Secondary outcomes

Eight studies [32, 36, 41, 43, 52, 55, 63, 71] included qualitative data about experiences. Participants reported positive experiences and high levels of satisfaction with the respective interventions. Six of these interventions, (Australia *n* = 5, UK *n* = 1), combined a midwifery model of care with a community-centred service and their recurring themes included trust, continuity of midwifery care, family-centred approaches, clarity of information shared and culturally appropriate care. The UK study [71] which was centred around an initial accommodation centre, reported negatively on the accommodation centre and its facilities rather than care provided.

Lower rates of low birth weight were reported in the intervention groups [43, 52, 59], however not all were statistically significant findings [34, 41, 74]. Preterm birth rates were also notably lower [34, 43, 45, 52, 59, 74]. Intervention group were more likely to have non-instrumental vaginal birth and have lower rates of caesarean section, along with lower rates of epidural analgesia use [43, 45]. In the intervention groups there were higher rates of first-trimester initial appointments and higher rates of antenatal care coverage [34, 41, 45, 52, 59, 74]. However, one study [43] had lower rates of timely first-trimester attendance due to delays in processes, furthermore, inadequate referral pathways meant some women were not allocated to eligible services. Breastfeeding rates were higher in intervention groups [32, 45, 52, 75].No data were reported regarding immunisations in the community-centred services interventions.

#### Patterns in countries

All community-centred service interventions were delivered either in the community setting solely or in combination with the hospital setting. The Australian interventions were predominantly in partnership with the community and frequently included a person of the same Aboriginality to deliver the service, such as a midwife, health worker, peer supporter or obstetrician. Two Australian studies combined all three interventions [43, 59] and targeted Aboriginal communities. Both studies provided midwifery-led continuity of care to women in their community setting and were delivered in partnership with the community health services. One Canadian [63], one Chilean study [32] and one USA study [60] combined interdisciplinary care with community-centred services and primarily targeted those living in deprivation and those with substance misuse issues. They both incorporated a range of practitioners, including physicians, paediatricians, counsellors, and substance misuse services, and were accessible in the local community to target hard-to-reach communities.

## Discussion

**Fig. 3:**
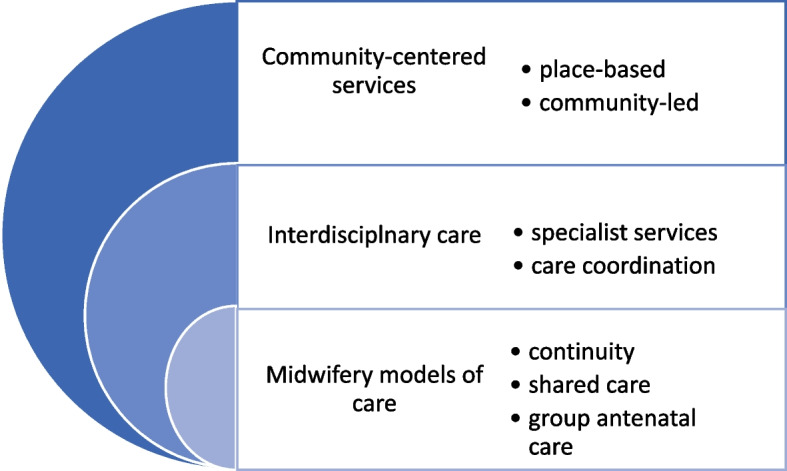
Stacked Venn diagram illustrating intervention types

This review systematically gathered and analysed the available evidence related to targeted health and social care interventions that have potential to reduce maternal and infant health inequalities in high-income countries. This review found 46 studies, that met the inclusion criteria, from a range of six high-income countries (Australia, Canada, Chile, Hong Kong, UK and USA) spanning from 1981 to 2021, using a variety of study designs. Interventions were implemented at different stages of maternity care including the antenatal, intrapartum and postnatal period. Some study interventions included a comparator in the form of a control group, retrospective clinical data, or national data whereas other studies had no comparator. Intervention types varied within countries/settings and between countries however, three principal intervention types were identified: midwifery models of care, interdisciplinary care and community-centred services. The intervention groups formed an order (see Fig. 3) based on the level of intervention but also, spontaneously, based on how frequently they were reported in this review.

The primary outcomes demonstrate that maternal, perinatal and/or neonatal mortality were positively impacted in some instances however they were often not statistically significant due to methodological limitations. Secondary outcomes were positively associated with interventions such as earlier access to care, more than four antenatal visits coverage, mode of birth (decreased caesarean-section, and increased vaginal birth), use of postnatal contraception, increased breastfeeding rates. However, statistical significance of results was sporadic or not consistently reported so difficult to establish causality.

This review focused on interventions with a clinical component of care to identify which models and programmes of care have been tested. However, the review did not include interventions with strong existing evidence for vulnerable populations, such as, family-nurse partnerships [26] and social support [27, 28] as it would not add to the body of evidence. Yet, it did include continuity of care and group antenatal care because there is not a good quality evidence available specifically for women at disproportionate risk of health inequalities.

The findings of this review suggest that existing interventions can help mitigate health inequalities in at risk populations and these are adaptable in different HIC contexts. For example, in settings where care from a midwife is not the standard it can help improve women’s experiences of maternity care and encourage early attendance and increase antenatal coverage. Furthermore, in settings where midwife-led care is the standard, continuous continuity from a midwife or team of midwives can also improve women’s experiences and feelings of satisfaction. When continuity is not provided throughout the maternity journey it leads to lower levels of satisfaction. This demonstrates that the professional group providing care is of importance to women, as it facilitates relationship building. Additionally, this review has also identified that care from those of similar background, as well as care in a group with those in similar situations (particularly adolescence) are important to women and helps relieve feelings of isolation. Overall, care from a midwife, or similarly qualified, is shown to positively impact primary or secondary outcomes in HICs and is therefore an important intervention to consider.

Women at higher risk of inequalities often require input from multiple agencies and clinical professionals [6]. This review found that input of specialist services, in coordination with maternity care, such as counselling for substance misuse, can be an effective intervention to improve engagement and utilisation of primary health services, and promote healthier choices. The findings also emphasise that for women requiring multiagency support, care coordinated by a midwife can facilitate improved experiences and overall quality of care.

This review recognised that community-based interventions, when combined with midwifery models or interdisciplinary care, can maximise the benefits of the intervention to promote health equality. Place-based care in the community is known to improve outcomes [21, 76] and this review supports this. Furthermore, this review adds to the evidence of social support but more specifically support within communities and community-controlled health services that are culturally specific.

The strength of this review is that it systematically searched a breadth of literature to identify the maximum number of studies across HICs with targeted interventions. This review also considered key primary and secondary outcomes to fully understand the potential impact of interventions. The limitations of this review is that the methodologically quality of studies included varied, yet no study was excluded based on their quality assessment in accordance with MMAT recommendations [29].

## Conclusion

Health and social models and programmes of care are complex interventions. This review identified that existing targeted interventions are overall positively associated with improved outcomes. However, they are of varying statistical significance, and impact across countries and contexts. Importantly, this review identified that multi-interventional approaches could enhance a targeted approach for those disproportionately at risk of health inequalities and experiencing multiple health and social disadvantages. Including community-centred approaches that are place-based and/or combined with hospital-based care can enhance accessibility, earlier engagement, and increased attendance. Midwife-led care is highly reported across settings and this review highlights that the holistic nature of midwifery care is highly valued by at risk women and can better facilitate care coordination for women with complex multiagency needs. Further to this, Australian based studies demonstrate that it is valuable to indigenous and minority ethnic groups to include health workers (midwife, support workers or health officers) from similar backgrounds to the local community as this enhances culturally competent care.

The findings of this review are applicable in HICs and their distinctive contexts, including variations in health financing and populations at disproportionate risk of health inequalities. This review can help inform policy makers understand which outcomes are positively associated with certain intervention types and recognise how to enhance already existing interventions which will fit within their health systems. This review did not intend to evaluate the interventions and nor would the included studies allow that, because they did not all consistently report the same level of detail. Future research should include detail of the intervention and theories of change so we can better understand how these interventions were implemented, mechanisms underlying the outcomes and whether interventions were delivered as intended. Future research would also benefit from comparative statistical analysis of the intervention and control groups for better interpretation of results. It will also be beneficial to research effectiveness of interventions separately from studies reporting experiences alone.

## Supplementary Information

**Additional file 1.** PRISMA-E Checklist.

**Additional file 2.** SPICE framework.

**Additional file 3.** Inclusion-Exclusion criterion.

**Additional file 4.** Outcome definitions.

## Data Availability

All related files have been attached to the submission as appendices or supplementary files.
