# Supplementary material for: Targeted health and social care interventions for women and infants who are disproportionately impacted by health inequalities in high-income countries: a systematic review
Source: Int J Equity Health. 2023 Jul 11;22:131. doi: 10.1186/s12939-023-01948-w (PMC10334506; doi:10.1186/s12939-023-01948-w)
Supplement: Supplementary file 2 — Additional file 2. SPICE framework. [file 12939_2023_1948_MOESM2_ESM.docx]

#### Appendix 2: SPICE framework

| **Keyword 1**  **(Setting)**  *Where?* |  | **Keyword 2 (Perspective)**  *For whom?* |  | **Keyword 3 (Intervention)**  *What?* |  | **Keyword 4 (Comparison)**  *What else?* |  | **Keyword 5**  **(Evaluation)**  *Result/How well?* |
| --- | --- | --- | --- | --- | --- | --- | --- | --- |
| High-income countries | **AND** | Childbearing women | **AND** | Health and/or social care intervention | **AND** | Health outcomes | **AND** | Health inequality |
| **Search term keyword 1** |  | **Search term keyword 2** |  | **Search term keyword 3** |  | **Search term keyword 4** |  | **Search term keyword 5** |
| High-Income or High-Income Countr* or High Income or High Income Countr* or  HIC or Andorra or Greece or Palau or (Antigua and Barbuda) or Greenland or Panama or Aruba or Guam or Poland or Australia or Hong Kong or Portugal or Austria or Hungary or Puerto Rico or Bahamas or Iceland or Romania or Bahrain or Ireland or Qatar or Barbados or Isle of Man or San Marino or Belgium or Israel or Saudi Arabia or Bermuda or Italy or Seychelles or British Virgin Islands or Japan or Singapore or Brunei Darussalam or Republic of Korea or South Korea or Saint Maarten or Canada or Kuwait or Slovakia or Cayman Islands or Latvia or Slovenia or Channel Islands or Liechtenstein or Spain or Chile or Lithuania or (Saint Kitts and Nevis) or Croatia or Luxembourg or Saint Martin or Curacao or Macao or Sweden or Cyprus or Malta or Switzerland or Czech Republic or Mauritius or Taiwan or Denmark or Monaco or (Trinidad and Tobago) or Estonia or Nauru or (Turks and Caicos) or Faroe Islands or Netherlands or United Arab Emirates or Finland or New Caledonia or United Kingdom or UK or France or New Zealand or United States or USA or French Polynesia or Northern Mariana Island or Uruguay or Germany or Norway or Virgin Islands or Gibraltar or Oman |  | Childbearing or Woman or Women or Female or Pregnant or Pregnan* |  | Maternity care or Maternal welfare or Maternal health or Maternal healthcare or Maternity Care or Maternal service* or Obstetric* or Midwi* or Birth Attendan* or Skilled Birth Attendan* or Traditional Birth Attendan* or Health Service* or Healthcare Service* or Service* or Health System* or Healthcare System* or System* or Intervention* or Programme* or Program* or Project or Health Polic* or Health Promotion or Reproductive Service* or Reproductive Health* or Contraception or Family planning service or Antenatal care or Prenatal care or Prenatal visits or Intrapartum Care or Delivery care or Postnatal care or Postnatal visits or Facility based delivery or Universal Healthcare or Universal Health Coverage or Universal Care or Safe motherhood or (Social, behavioural and community engagement intervention) or SBCE or Home visits or Interpersonal communication or Group ICP or (Social Media and m-health) or Social marketing or Demand size financing or Community based health insurance or Community mobilisation* or Interpersonal counselling or Community participation or social accountability or community engagement |  | Antenatal cover* or Contraception or Birth Control or Abortion or Family Plan* or Maternal Mortalit* or Maternal Morbidit* or Maternal Outcome or Perinatal Mortalit* or Perinatal Morbidit* or Perinatal Outcome or Neonatal Mortalit* or Neonatal death* or Stillbirth* or Neonatal Morbidit* or Neonatal Outcome or Infant Mortalit* or Infant Morbidit* or Infant Outcome or Child mortality or Child morbidity or Newborn survival or Prematur* or Preterm or Kangaroo Mother Care or Skin to Skin or Low birth weight or Malnutrition or Breastfeeding or Vaccination or Infection or Congenital abnormalities |  | Health Inequal* or Health inequit* or Equality or Inequalit* or Equity or Inequit* or Disparit* or Healthcare Disparit* or Health Status Disparit* or Antenatal cover* or Access or Quality of care or Satisfaction or Trust Engagement or Knowledge or Attitudes or Care seeking behaviour or Communication |
