# Supplementary material for: Targeted health and social care interventions for women and infants who are disproportionately impacted by health inequalities in high-income countries: a systematic review
Source: Int J Equity Health. 2023 Jul 11;22:131. doi: 10.1186/s12939-023-01948-w (PMC10334506; doi:10.1186/s12939-023-01948-w)
Supplement: Supplementary file 3 — Additional file 3. Inclusion-Exclusion criterion. [file 12939_2023_1948_MOESM3_ESM.docx]

#### Appendix 3: Inclusion-Exclusion criterion

|  | **Inclusion** | **Exclusion** | **Justification/Explanation** |
| --- | --- | --- | --- |
| Date of publication | All | No restriction | The purpose of the review is to synthesise all available research, to date. |
| Study design | All | No restriction | Primary research papers contained in literature reviews, which meet the criterion, will be included directly in the full-text screening. |
| Setting | High-income countries (see box 1) | Low- or middle-income countries | Country health contexts vary however, HIC’s are more likely to have an established “standard care” pathway and fewer resource or financial limitations. |
| Language | All | No restriction |  |
| Population | Childbearing women (AN, IP, PN periods), newborns and infants up to one year of age who are disproportionately impacted by health inequalities | Non-childbearing women  Children over the age of one  Men  Healthcare Professional’s  Sites (geographic or hospital) that recruit from disadvantaged areas but do not target individual characteristics of women | The aim of the review requires the population group to be limited to childbearing women, newborns and infants up to the age of one.  Women/babies that are known to be disproportionately impacted by health inequalities and have complex social risk factors. |
| Intervention | Any health or social care service intervention, that is a programme/package of care e.g., placed based models, specialist care or programmes, home visiting interventions, mobile health clinics, case management, enhanced AN care programmes, specialist clinics, continuity of carer, peer support that is provided by health/social care.  **This should include an element of clinical care as part of the programme/package of care, that is different from “standard care”.** | Standalone interventions (clinical or non-clinical) (e.g., testing, supplements, voucher, leaflet).  Interventions that are not part of a programme/package of care or are an adjunct to standard care pathway (e.g., educational class, health promotion initiative, standard care pathway).  Non-health/social care interventions (e.g., lay/peer support workers, charity initiatives, neighbourhood projects, third sector programmes that do not provide clinical care).  Interventions which have current Cochrane reviews/trials, e.g., family nurse partnership and social support | This review defines a programme/package of care as a service, or combination of services, provided to patients/populations by health or social care services. The purpose of the review is to identify interventions designed to improve health and reduce health inequalities, compared to standard maternity care.  Third sector interventions can be included if they are integrated with a health or social care service e.g., community health advocates.  Interventions types will be assessed based on the methodology of the studies and if there is clear identification of interventions implemented.  An intervention described as a programme/package by the author/services will not be automatically included. The described intervention must meet the inclusion criteria and definition of programme/package of care as outlined in this review. Two authors will assess this based on the paper’s methodology and reach consensus. Conflicts will be discussed with the wider team.  Interventions that are well established and highly reported will be excluded to avoid replication. |
| Outcome | Primary:   1. Infant mortality 2. Perinatal mortality 3. Maternal mortality   Secondary:   1. Experience/ satisfaction 2. Family planning 3. Breastfeeding 4. Immunisation 5. Low birth weight 6. Preterm birth 7. Analgesia use 8. Mode of birth (spontaneous vaginal delivery and caesarean section) 9. Antenatal care coverage (4 visits) 10. Access to care 11. Quality of care | Outcomes that are not health or social care indicators.  No outcome data. | Papers that describe a health or social care service interventions but do not present any outcomes will be excluded as this does not meet the purpose of the review e.g., service modelling, theoretical papers.  Protocol papers with ongoing studies will be identified in this reviews appendices. |
| Evaluating/  Indicator | Inequality; inequity; disparity; health status; variation; quality of care; access; satisfaction; engagement; knowledge; attitude; communication. |  |  |
