# Supplementary material for: Targeted health and social care interventions for women and infants who are disproportionately impacted by health inequalities in high-income countries: a systematic review
Source: Int J Equity Health. 2023 Jul 11;22:131. doi: 10.1186/s12939-023-01948-w (PMC10334506; doi:10.1186/s12939-023-01948-w)
Supplement: Supplementary file 4 — Additional file 4. Outcome definitions. [file 12939_2023_1948_MOESM4_ESM.docx]

#### Appendix 4: Outcome definitions

|  |  |
| --- | --- |
| Primary outcomes |  |
| Outcome | Definition |
| Maternal mortality | Death in pregnancy, childbirth and puerperium. |
|  | Direct deaths – resulting from obstetric complications of the pregnancy. |
|  | Indirect death – resulting from previous exiting disease or disease aggravated |
| Perinatal mortality | Stillbirths (from 22 weeks) and neonatal death (within the first 28 days of life) |
| Infant mortality | Infant death before reaching 1 year of age |
|  |  |
| Secondary outcomes |  |
| Outcome | Definition |
| Experiences and satisfaction | As defined by authors |
| Family planning | As defined by authors |
| Breastfeeding | Initiation of breastfeeding |
| Immunisation | Use of vaccinations |
| Low birth weight | Birth weight less than 2500g |
| Preterm birth | Live birth before 37 weeks of pregnancy |
| Analgesia use | As defined by author |
| Spontaneous vaginal birth | Birth without any intervention |
| Caesarean section | Surgical intervention to deliver baby |
| Antenatal care coverage | Minimum 4 visits |
| Access to care | Gestational age at booking, financial assistance/insurance, distance and outreach |
| Quality of care | Referrals, timeliness, length of visits |
